# A distinctive DNA methylation pattern in insufficient sleep

**DOI:** 10.1038/s41598-018-38009-0

**Published:** 2019-02-04

**Authors:** Alexandra Lahtinen, Sampsa Puttonen, Päivi Vanttola, Katriina Viitasalo, Sonja Sulkava, Natalia Pervjakova, Anni Joensuu, Perttu Salo, Auli Toivola, Mikko Härmä, Lili Milani, Markus Perola, Tiina Paunio

**Affiliations:** 1 Department of Public Health Solutions, Genomics and Biomarkers Unit, National Institute for Health and Welfare, PO Box 30, FI-00271 Helsinki, Finland; 2 Department of Psychiatry, University of Helsinki and Helsinki University Central Hospital, PO Box 590, FIN-00029 HUS Helsinki, Finland; 3 Work Ability and Working Career, Finnish Institute of Occupational Health, PO Box 40, FI-00032 Työterveyslaitos Helsinki, Finland; 4 Finnair Health Services, HEL-IF/67, FI-01053 Finnair, Finland; 5 Estonian Genome Center, Institute of Genomics, University of Tartu, Tartu, 51010 Estonia; 6 Diabetes and Obesity Research Program, University of Helsinki, PO Box 63, FI-00014 Helsinki, Finland

## Abstract

Short sleep duration or insomnia may lead to an increased risk of various psychiatric and cardio-metabolic conditions. Since DNA methylation plays a critical role in the regulation of gene expression, studies of differentially methylated positions (DMPs) might be valuable for understanding the mechanisms underlying insomnia. We performed a cross-sectional genome-wide analysis of DNA methylation in relation to self-reported insufficient sleep in individuals from a community-based sample (79 men, aged 39.3 ± 7.3), and in relation to shift work disorder in an occupational cohort (26 men, aged 44.9 ± 9.0). The analysis of DNA methylation data revealed that genes corresponding to selected DMPs form a distinctive pathway: “Nervous System Development” (FDR *P* value < 0.05). We found that 78% of the DMPs were hypomethylated in cases in both cohorts, suggesting that insufficient sleep may be associated with loss of DNA methylation. A karyoplot revealed clusters of DMPs at various chromosomal regions, including 12 DMPs on chromosome 17, previously associated with Smith-Magenis syndrome, a rare condition comprising disturbed sleep and inverse circadian rhythm. Our findings give novel insights into the DNA methylation patterns associated with sleep loss, possibly modifying processes related to neuroplasticity and neurodegeneration. Future prospective studies are needed to confirm the observed associations.

## Introduction

Lack of sleep and sleep disturbances are extremely common in the general population^1,2^. According to the population-based surveys conducted in Finland, up to one-third of the general adult population experiences occasional difficulties in sleep^2^ and almost one-fourth reported sleeping 6 hours or less per night^2,3^. Chronic lack of sleep affects health; specifically, it increases the risks of cardio-metabolic disorders such as type 2 diabetes and cardiovascular diseases, as well as mental disorders^4^. The symptoms of insomnia have become widespread, especially in the working population: in 2007, a report based in Finland revealed that insomnia-related symptoms were frequent among 9% of workers, and occasional in up to 45.3%^3^. Night and early morning shift work is a common source of insufficient sleep, since it leads to circadian misalignments, misbalances homeostasis, and truncates total sleep time by 1–4 hours^5^. The detrimental effects of shift work on sleep can result in shift work disorder (SWD) – a medical condition characterized by complaints of excessive sleepiness and/or insomnia as primary symptoms, accompanied by a reduction in sleep duration during the working period^6^. It is a common condition affecting over one-third of shift workers^7–9^.

The effect of sleep deprivation on the transcriptome and methylome has previously been studied both in experimental animal models and in selected samples^10–15^. Sleep deprivation induces notable changes in the brain transcriptome of rats, affecting protein synthesis, synaptic plasticity, and metabolism^10,16^. For example, Archer *et al*.^17^ found a reduction of rhythmic transcripts and major changes in the transcriptome once sleep was mistimed. Simply missing a single night’s sleep alters both the transcriptional and the DNA methylation profiles of core circadian clock genes^13^. Indeed, Bhatti *et al*.^18^ found a significant decrease in average methylation among the nightshift workers compared to the methylation profiles of dayshift workers.

Despite the extensive ‘omics’ studies conducted in human cohorts during the last decades, the biological mechanisms underlying the negative consequences of chronic lack of sleep in workers are not fully understood and the inter-individual variation of such consequences is known to be quite large. Our previous work^19^ focused on intrinsic genetic risk factors for intolerance to shift work, and found an association between job-related exhaustion and a variant downstream of the melatonin receptor 1A gene with a proposed mechanism of changes in DNA methylation at the gene promoter, when exposed to the risk environment (shift work).

In this study, we investigated DMPs in blood leukocytes that reflect the systemic stress triggered by insufficient sleep in two complementary samples of cases and controls from (i) individuals selected from a community-based sample (DILGOM, a sub-study of the population-based FINRISK) and from (ii) an occupational cohort of shift workers (Airline). In (i), cases were selected based on their self-reported evaluation of sleep insufficiency, meaning seldom or hardly ever sleeping enough. In (ii), cases were selected based on the presence of SWD symptoms specifically during shift work periods: symptoms of insomnia, sleepiness, and objectively measured reduction in total sleep time.

## Results

The DILGOM sample included a total of 517 unrelated individuals from Helsinki, Finland, recruited for the Dietary, Lifestyle, and Genetic determinants of Obesity and Metabolic syndrome study in 2007, with extensive information on traits and lifestyle factors. For our study, we focused on the FINRISK survey question “Do you, in your opinion, sleep enough?” Based on the answer, we dichotomized our study group into the cases and controls (See Materials and Methods section “Study samples” for detailed information).

We first investigated the effect of age and gender on DNA methylation in the complete DILGOM sample (N = 517). Age and gender were found to strongly affect the DNA methylation (Supplementary Fig. S1): 25.6% (122,721/479,954) and 7.9% (37,780/479,954) of all CpGs showed FDR-corrected *P* values < 0.05 for age and gender effect, respectively.

Thus, considering the reported effect of age and gender on the primary phenotype of our study (insufficient or disturbed sleep^20,21^), and, furthermore, the fact that most (81%) of the participants in the Airline sample were < 50 (n = 26), we focused on men < 50 years old in the DILGOM sample (n = 79) in the subsequent analyses. However, as age has an important effect on DNA methylation, it was included in all analyses as a covariate.

### Methylome-wide analysis of DILGOM and Airline

In order to detect DNA methylation modifications in relation to insufficient sleep, we performed epigenome-wide association studies in two complementary samples: a population-based DILGOM sample (n = 79, age = 39.3 ± 7.3, from a sample total of N = 517) and an occupational Airline sample of shift workers (n = 26, age = 44.9 ± 9.0) by comparing genome-wide methylation between cases and controls in each sample.

We identified 14,487 (DILGOM) and 19,303 (Airline) DMPs with uncorrected *P* values < 0.05. None of the sites survived an FDR-corrected threshold of 5%. After comparing the results for both groups, we identified 399 sites that were common to both samples and with the same direction of methylation in DILGOM and Airline (hyper- or hypomethylated sites), with 327 DMPs annotated to 317 genes. The majority 248 (78%) of the CpG sites were hypomethylated in cases compared with the controls (Supplementary Table S2).

### Pathway analyses and database search of the overlap set

In order to identify the affected biological pathways, we used several freely available online resources (GoMiner, Panther, g:Profiler) and one commercially available pathway analysis tool, IPA. The comparison of results revealed two major groups of pathways – nervous system development (NSD) and cellular processes, with NSD (GO:0007399) common to all pathway tools (FDR-corrected *P* values: 0.0013, GoMiner; 0.0137, Panther; 0.00131, g:Profiler; uncorrected *P* value 1.54E-5, IPA). NSD comprised 92 DMPs annotated to 89 genes after duplicates were removed, in all tools (see Supplementary Table S3).

As an alternative strategy to search for the implications of the DMPs, we investigated the phenotype associations related previously for the set of 317 genes common to both groups, using the Ensembl genome browser (Human GRCh38.p10). Of these, 59 had no information about phenotype association; 182 were related to non-specific pathological conditions; 19 were associated with various sleep phenotypes (including circadian rhythms); 50 were associated with a genetic syndrome. Since 13 out of 50 genetic syndromes included some type of disturbances in visual processing (mostly retinal abnormalities), we performed an additional check of all 317 genes and discovered 8 genes that are associated with disturbances in visual processing (see Supplementary Table S4). Altogether, 79 out of 317 genes were associated with a specific syndrome, sleep problems, or visual disturbances.

**Figure 1:**
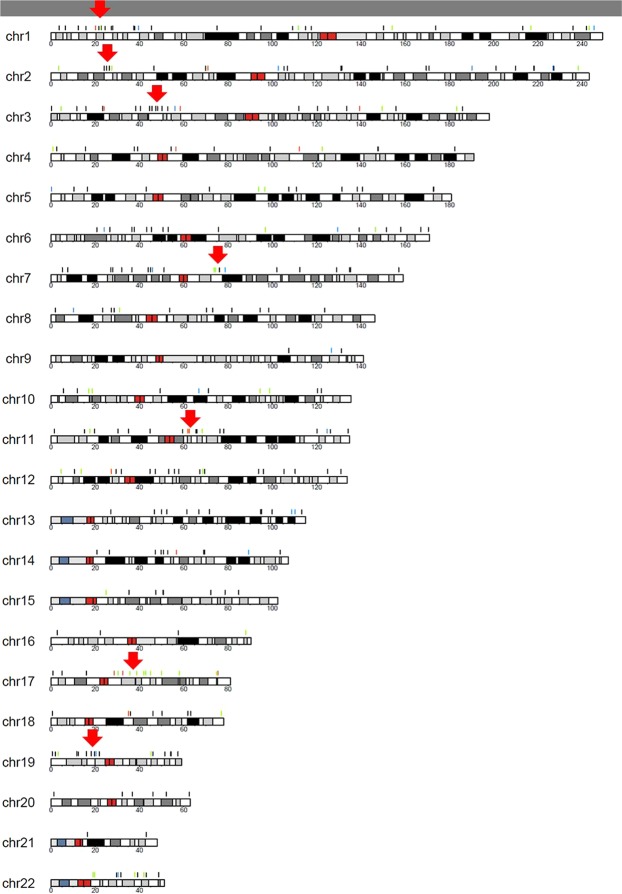
Common 317 DMPs presented in a karyoplot. Arrows indicate clusters of DMPs in various locations. Marked in colors are DMPs corresponding to genes associated with sleep (blue), both sleep and disturbances in visual processing (green), and others (black).

**Figure 2:**
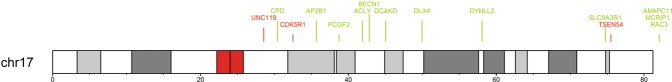
Genes associated with Smith-Magenis syndrome (green) and disturbances in visual processing (red), located on chromosome 17 of the human genome, corresponding to DMPs (Rat Genome Database and Ensembl database). Numbers under the chromosome indicate coordinates in bp.

The 317 genes were plotted in a karyoplot (Fig. 1), which revealed clusters of DMPs at various chromosomal locations. One of these clusters comprised 15 DMPs of genes on chromosome 17. Of these 15 genes, 12 have previously been associated with Smith-Magenis syndrome (SMS) in rodents or humans according to the rat genome database, and 3 genes are related to various retinopathies in animals and human patients (Fig. 2). The karyoplot identified several other specific regions of interest, including 1p36.12 (4 DMPs), 2p23.3 on chromosome 2 (4 DMPs), 3p21.31 on chromosome 3 (6 DMPs), 7q11.23 (4 DMPs), 11q13.1 (3 DMPs), and 19p12 (3 DMPs).

### DNA methylation and gene expression

We further investigated whether methylation at the identified DMPs correlates with the expression level of the corresponding gene. We performed correlation analyses of M-values with corresponding gene expression levels for 327 DMPs/317 genes/390 RNA transcripts. The methylation levels of 32 (10%) DMPs were correlated with the gene expression levels in DILGOM at *P* < 0.05 (Supplementary Table S5). None of the results were significant according to the Bonferroni-corrected threshold of 0.05/400 after multiple testing corrections. However, we identified an excess of DMPs whose methylated levels were correlated with the corresponding levels of gene expression at *P* < 0.0001 (observed 5 vs expected 1) and *P* < 0.01 (observed 12 vs expected 4). Four of these 32 sites found in DILGOM (cg27013755 *BANP*, cg03193489 *TERF1*, cg11214001 *CAST*, and cg14603227 *NAV2*) showed correlations of DNA methylation levels with RNA expression levels also in the Airline sample at *P* < 0.05.

### Correlation analysis of M-values with sampling time

Since expression levels of some of the genes identified earlier have previously been found to fluctuate according to circadian time^17^, we studied the correlation of the methylation values with the time of sample collection. In DILGOM, we found M-values of 6/399 DMPs, to correlate with the sampling time at *P* < 0.05 (not significant after multiple testing corrections, see Supplementary Table S6). In Airline, we found 14 DMPs whose M-values correlated with the sampling time at *P* < 0.05 (not significant after multiple testing corrections, see Supplementary Table S7). None of these DMPs are identical to the ones found significant in the DILGOM sample.

## Discussion

In this study, we identified suggestive deviations in DNA methylation of blood leukocytes associated with subjective sleep insufficiency in a population cohort and in an occupational shift work sample. We found a distinctive pattern of DMPs among individuals suffering from sleep disturbances. This pattern consisted of 399 DMPs, out of which 327 were annotated to genes that showed an enrichment of associations with the nervous system development pathway (NSD).

According to the Gene Ontology database, the NSD pathway is defined as the process of either the maturation of nervous tissue or its progression over time. It includes the processes of neurogenesis, regulation of nervous system development, synapse maturation, nerve development, and others. Our findings are in agreement with previous studies, in which DNA methylation differences induced by sleep disturbances were observed in pathways involved in synaptic plasticity and neuritogenesis^12^. However, earlier studies involved the analysis of DNA methylation in the brain tissue of rodents, whereas our study used blood samples from humans. Since the lack of sleep has a systemic effect on the human body, we suggest that blood samples are informative source for revealing systemic changes triggered by insufficient sleep.

The analysis of genome location of 317 genes indicated several clusters, most prominent on chromosome 17. Interestingly, 12 of these 18 genes located on chromosome 17 relate to SMS in rodents or humans (Fig. 2). SMS [MIM 182290] is a rare genetic disorder affecting 1 in 25 000 individuals, and in most cases is caused by a 3.7 Mb interstitial deletion in the short arm of chromosome 17. SMS affects the whole body, with disturbed sleep being one of the prominent symptoms (75–100% of cases according to Greenberg *et al*.^22^). Thus, the affected individuals have difficulty falling asleep at night and staying awake during the day, due to the inverted circadian rhythm of melatonin^22^. Some SMS patients have noted the absence of rapid eye movement (REM) sleep^23^. The mutations in *RAI1*, located in the 17p11.2 locus are known to be responsible for the inversion of the melatonin cycle^24^. However, not all SMS patients with *RAI1* mutations or deletions experience sleep disturbances^25^, hence the SMS phenotype may be achieved by complex rearrangements of chromosome 17 that also involve its long arm^26,27^. The observation of DNA methylation changes in the group of genes related to SMS requires further investigation, possibly in occupational cohorts of shift workers, since shift work can lead to circadian misalignments and sleep loss^5^. Our findings nevertheless suggest that this region on chromosome 17, earlier linked to SMS, may also play an important role in regulation of sleep and circadian rhythm in the general population via epigenetic regulatory mechanisms.

In chromosome 3p21.31, we identified a cluster of 6 genes, of which 5 (*LIMD1*, *SCAP*, *CDC25A*, *SLC38A3*, and *NEK4*) are known to be associated with cell responses to stresses such as hypoxia (*LIMD1*^28^ and *SCAP*^29^), UV-induced DNA damage (*CDC25A*^30^), potassium restriction (*SLC38A3*^31^) or DNA damage (*NEK4*^32^). Several earlier studies have highlighted the association of sleep deprivation with cellular stress^33,34^. Based on our results, we suggest that this association might also involve epigenetic mechanisms.

In 7q11.23, there was a small group of 4 genes including 3 genes related to anxiety and autism (*STX1A*^35^, *GTF2IRD1*^36^, and *MAGI2*^37^). Finally, on chromosome 19, we discovered a group of genes possibly involved in regulation of transcription: *ZNF441*, *ZNF709*, *ZNF506*, *ZNF826*, and *ZNF43*. In our set of 317 genes there were 11 zinc finger transcription factors with affected methylation levels (9 hypo- and 2 hypermethylated), and 5 of them are located on chromosome 19. Possibly, loss of methylation of transcription factors induced by sleep disturbances contributes to the overall change in gene expression^17,38^.

The analysis of phenotypic associations of the overlap set of common genes revealed a large number of genes related to various disturbances in visual processing (31 genes, see Table 1), many of which are involved in retinal light transmission. For example, *UNC119* is specifically expressed in the photoreceptors in the retina, and plays a role in photoreceptor neurotransmitter release through the synaptic vesicle cycle^39^. *UNC119* is also located on chromosome 17, together with *TSEN54* and *CDK5R1*, known for sight-threatening retinopathy in type 2 diabetes and disturbed retinal morphology, respectively. This finding is interesting considering the central role of light as an external zeitgeber for circadian rhythm.

**Table 1:** Genes corresponding to DMPs associated with disturbances in sleep, visual processing, and both sleep and visual processing.

Genes associated with sleep disturbances	Ensembl Phenotype
*ESRRG, KIF26B, HIBCH, COL4A2, ZN826*	Sleep duration (daytime naps)
*RHBDD1, MSRA, FOXN3, ERC2, NR1D2*	Circadian phenotypes, chronotypes
*NRSN1*	Abnormal sleep behavior
*SLC9A2, CTNNA3, USH1C*	Awakening after sleep onset
*NRGN, GRIN2B*	Sleep deprivation
*ARHGAP18, LMX1B*	Sleep onset
*MAGI2*	Sleep offset
*HEYL, OSM, AHRR*	Breathing rate during sleep
*MCF2L2, WWTR1, VIM*	Behçet syndrome (sleep disturbances)
*ACLY, ANAPC11, AP2B1, BECN1, CPD, DCAKD, DLX4, DYNLL2, FAM195B, PCGF2, RAC, SLC9A3R1*	Smith-Magenis syndrome (melatonin cycle inversion)
**Genes associated with disturbances in visual processing**	***Ensembl Phenotype***
*IFT172, FTH1, ROM1, UNC119, MAPRE2, USH1C*	Retinitis pigmentosa
*SDCCAG8, LRP5, USH1C*	Photoreceptor degeneration
*STK38L, OTX2*	Retinal dystrophy
*UNC119, BBS12*	Cone-rod dystrophy
*VIM, NR1D2*	Cataract
*LMX1B*	Glaucoma
*UNC119, CDK5R1, TSEN54*	Retinopathy
*ALPK1, NR1D2*	Iris morphology abnormalities
*VAX2, RBP1, PAICS, CDK5R1, HEYL, ERC2, MCF2L2, WWTR1, PLA2G5, GTF2IRD1*	Retinal morphology abnormalities
**Genes associated both with sleep and disturbances in visual processing**	**Ensembl Phenotype**
*MCF2L2, WWTR1*	Poor sleep quality, retinal abnormalities
*HEYL, AHRR*	Breath rate during sleep, abnormal retina morphology
*ERC2*	Morning vs. evening chronotype, daytime napping duration, rest duration at nights, retina morphology
*BBS12*	Rod-con dystrophy, sleep latency
*GTF2IRD1, VIM*	Sleep disturbances, retinal changes, cataract
*LMX1B*	Sleep initiation and maintenance disorders, glaucoma, ocular hypertension
*USH1C*	Retinitis pigmentosa, retinal dystrophy, photoreceptor degeneration, wake after sleep onset
*GRIN2B*	Sleep deprivation, rats, ocular disruptions

In the overlap set of 317 genes, we discovered a group of 11 genes that are involved in both sleep and disturbances in visual processing (Table 1, last section). For example, variants in *ERC2* have been found to be significant in genome-wide association studies for chronotype and daytime sleep phenotypes^40,41^. *ERC2* was also found to be important for normal visual processing in mice, as its inactivation impairs synaptic encoding of visual stimuli^42^. Another example is *GRIN2B* – one of the subunits of the glutamate ionotropic receptor of NMDA type. In a study of rats^43^, those that were sleep-deprived had lower expression of *GRIN2B* in the hippocampus, leading to hippocampal synaptic impairment. NMDA receptors also play a role in photoreceptor functioning, as described in the study of rat models with retinitis pigmentosa^44^.

The majority of the DMPs (76%) were relatively hypomethylated, suggesting that a lack of sleep may be associated with a loss of methylation, consequently leading to systemic alterations of gene expression. This finding is concordant with a study of nightshift workers^18^, which revealed decreased DNA methylation in the blood of men lacking sleep. However, it is noteworthy that the effect of hypomethylation on transcription depends on the location of a given CpG (i.e. promoter region, gene body), which makes it difficult to draw any conclusions about increased or decreased gene expression.

A few limitations in this study warrant consideration. The first is the small sample size of the studied groups. Both samples sizes were not large enough to generate sufficient statistical power; however, combining results from the two independent cohorts was a major strength of our study. Second, in order to diminish the heterogeneity, based on our preliminary analysis (Supplementary Fig. S1), we focus only on men of a certain age in both studied samples. The third limitation is the use of blood samples for the study of sleep. Methylation is tissue-specific, so our findings must be interpreted with caution. However, disturbed sleep and circadian rhythm have systemic effects on the human body, as reported earlier^17^. Fourth, the phenotype data used in this study are based on self-reported sleep sufficiency, without objective measurements, which is a common limitation for many population surveys. Use of the self-reported criteria allowed us, however, to capture an essential part of the individual variability in the intrinsic need for sleep, which varies due to a number of factors, including genetic^45,46^. For example, the Airline control group, there were individuals who could sleep regularly for 6 hours or less, but did not complain about tiredness or symptoms of insomnia. Fifth, our study was cross-sectional, and future prospective studies are needed to establish the temporal relationship between sleep insufficiency and changes in DNA methylation. Finally, despite the homogeneity of the samples (men matched in age), there are, however, some important differences. The DILGOM respondents from the general population reported insufficient sleep due to various reasons, while the Airline group was dichotomized according to the symptoms of SWD specifically during shift work periods. In addition to the subjective experience of insufficient sleep, the cases in DILGOM reported also an increased amount of symptoms of insomnia, tiredness, and short sleep, while the diagnostic definition of SWD in the Airline sample included symptoms of insomnia, sleepiness, and reduction in total sleep duration. Thus, cases shared similarities at the level of sleep symptoms in these two complementary samples for insufficient sleep. It is, however, noteworthy that our study on men below 50 years of age cannot be generalized to wider or other populations.

## Conclusion

In conclusion, our findings suggest that there is a distinctive pattern of genes showing a diversity of epigenetic modifications in relation to subjective sleep insufficiency in men below 50 years of age. These differences related to compromised neuroplasticity and neurodegeneration (involving genes, such as *ERC2*, *MAGI2*, *CAST*, and *CDK5R1*) might be triggered by insufficient sleep. The clarification of the processes behind the observed association requires further investigation, both in general population-based samples or larger occupational cohorts, and in experimental data.

## Methods and Materials

### Study samples

#### Population-based study

Subjects were obtained from the DIetary, Lifestyle, and Genetic determinants of Obesity and Metabolic syndrome (DILGOM) study, which is an extension of the FINRISK 2007 study aimed at assessing risk factors for metabolic and cardiovascular diseases in the Finnish population^47^. The DILGOM general population subsample comprised 517 unrelated Finnish individuals from the Helsinki area, aged 25–74 years (54% females, age = 51.9 ± 13.8). Our study comparing DNA methylation for insufficient sleep in blood samples included only men aged 25–50 years, with available data on smoking status and alcohol consumption (n = 88). In order to select the cases for the present study, we used the FINRISK survey question “Do you, in your opinion, sleep enough?” as described in Aho *et al*.^38^. Individuals who answered “Seldom or almost never” comprised cases (n = 17), others were controls (answers “Yes, almost always” and “Yes, often” combined to one group, n = 62). Nine individuals answering “I cannot say” were excluded, leaving 79 men in total (age = 39.3 ± 7.3). In addition to the subjective report of sleep insufficiency, all cases reported at least one of the following sleep-related symptoms: symptoms of insomnia during the last month (“often or sometimes”), short sleep (sleep duration of 6 hours or less), or tiredness in the mornings (“rather” or “very tired”). Almost all cases (94%) reported two or three of these sleep-related symptoms. The majority of the DILGOM controls (84%) reported none or one of the above-mentioned sleep-related symptoms. The blood samples were collected between 7 a.m. and 1 p.m. (the exact time was recorded for each participant), after a fasting period of at least 10 hours.

#### Occupational shift work study

The occupational sample Airline included 42 workers aged 27–60 (21% females) from a Finnish airline company, whose shift work schedule included mornings (starting by 6 a.m.) and/or nights (at least 3 hours of work between 11 p.m. and 6 a.m.) in addition to evenings. The current study focused on 26 male shift workers (age = 44.9 ± 9.0) with DNA samples and available data on smoking status and alcohol consumption. SWD and non-SWD groups were defined using: (1) questions assessing the shift type and work day-specific symptoms of insomnia and sleepiness, and (2) a 3-week sleep diary and actigraphy monitoring to detect a work shift-related reduction of total sleep time, as required by International Classification of Sleep Disorders – Third Edition (ICSD-3) criteria^6^. To be deemed an SWD case (n = 17), a participant had to report symptoms of insomnia and/or sleepiness “often/continuously” related to a work shift only (i.e. not while on holiday), and had to have a reduced total sleep time. Male shift workers lacking significant insomnia and sleepiness symptoms constituted the non-SWD control group (n = 9). For the blood sample collection performed between 7 a.m. and 11 a.m., the participants had to be healthy and it should have been 7 days since the last infection episode occurred. The samples were never taken while leaving from a night shift. See Table 2 for detailed characteristics of the DILGOM and Airline participants.

**Table 2:** Characteristics of participants in the DILGOM and Airline groups.

	DILGOM		Airline	
	cases (n = 17)	controls (n = 62)	cases (n = 17)	controls (n = 9)
Age (SD), years	40.1 (7.5)	39.0 (7.3)	41.8 (8.8)	46.7 (6.7)
Sufficient sleep, self-report				
“Seldom or almost never”	17 (100%)	0	—	—
“Yes, almost always”	0	19 (31%)	—	—
“Yes, often”	0	43 (69%)	—	—
Shift work disorder status	—	—	17 (100%)	0
Smoking status				
“Never smoked”	9 (53%)	35 (56%)	5 (29%)	3 (33%)
“Quit”	5 (29%)	10 (16%)	9 (53%)	6 (67%)
“Current smoker”	3 (18%)	17 (28%)	3 (18%)	0
Alcohol consumption				
“At least once a month”	15 (88%)	52 (84%)	—	—
“Rarer than once a month”	2 (12%)	7 (11%)	—	—
“I quit alcohol”	0	3 (5%)	—	—
“I never consume alcohol”	0	0		
“Once a month or rarer”	—	—	0	0
“2–4 times per month”	—	—	9 (53%)	2 (22%)
“2–3 times per week”	—	—	5 (29%)	5 (56%)
“at least 4 times per week”	—	—	3 (18%)	2 (22%)
Data are mean (SD), n (%)				

DILGOM = DIetary, Lifestyle, and Genetic det erminants of Obesity and Metabolic syndrome.

### DNA methylation and gene expression

#### Infinium HumanMethylation450K BeadChip methylation measurements

In both study samples, the DNA extraction, CpG methylation, and quality control procedures were performed in the same manner. For DILGOM, DNA was extracted from whole blood samples and CpG methylation was performed using Infinium HumanMethylation450k BeadChip (Illumina, Inc., San Diego, CA, USA), as described in Karlsson Linnér *et al*.^48^. For Airline, DNA was extracted from whole blood samples using the same standard methods. DNA methylation was performed using Infinium HumanMethylation450K BeadChip. We conducted post-array processing and normalization for Airline in the same manner, as described in Karlsson Linnér *et al*.^48^. Briefly, raw intensity data files were preprocessed, and M-values (log2 ratio of the intensities of methylated probe vs unmethylated probe) were generated as described in the R package “minfi”^49^. Methylation values were corrected for background, followed by normalization with a subset quantile normalization approach (SWAN)^50^. After manual inspection of the control probe signals, no outliers were detected; no sex discrepancies were identified after checking the sex prediction samples. The quality control procedure removed the following probes: (a) probes with a low (<95%) detection rate at *P* < 0.01; (b) known cross-reactive probes^51^, (c) probes located on sex chromosomes; (d) probes containing a SNP either at the CpG interrogation or at the single nucleotide extension^51^. After these steps, 479,953 and 433,479 probes remained for the DILGOM and Airline samples, respectively.

#### Gene expression measurements

Gene expression data were extracted from Illumina HumanHT-12 Expression BeadChip (Illumina Inc., San Diego, CA, USA) for the DILGOM subsample of 79 men aged 25–50 and the Airline subsample of 12 male shift workers (7 cases with SWD, 5 controls with non-SWD). Gene expression detection and data processing, including quantile normalization, were conducted as described in Inouye *et al*.^47^.

### Analyses

#### Methylome-wide analysis

White blood cell proportions for DILGOM and Airline were estimated from methylation data by the Houseman method implemented in the ‘minfi’ package in R^52^. In the DILGOM sample, we first evaluated the effect of age and gender on methylation in the entire sample. The effect of sleep insufficiency on DNA methylation was only analyzed in men aged 25–50 years, as described in “Study samples”. The subjects were divided into two groups based on their self-reported sleep insufficiency (insufficient sleep status). In the Airline sample, subjects (27–60 years old) were divided into two groups based on the severity of the symptoms of SWD. For testing the correlation between each CpG site’s M-value and insufficient sleep or SWD status, we used a multiple linear regression model adjusted for age, smoking status, alcohol consumption, estimated white blood cell subtype proportions, and methylation array batch. Hypomethylation or hypermethylation was defined from the value of the beta coefficient in our regression model, with hypomethylation defined by a negative value. We applied a false discovery rate (FDR) Benjamini-Hochberg correction method for multiple testing to the results. Analyses were performed using R software (R = 3.3.0). Differentially methylated positions (DMPs) (*P < *0.05 cutoff level of significance) found common for both studied groups and having the same value (positive or negative) of the estimate in the regression model were annotated to genes, using Infinium HumanMethylation450k BeadChip annotation data.

#### Pathway analyses

To identify enriched gene ontology (GO) terms for the genes corresponding to DMPs identified in the previous step, we used GoMiner (https://discover.nci.nih.gov/gominer/index.jsp), Panther (http://www.pantherdb.org), and g:Profiler (https://biit.cs.ut.ee/gprofiler) online software tools. An unranked list of gene names served as the input and *P*  <  0.05 (after Benjamini-Hochberg multiple testing correction) was used to indicate statistical significance. We also used QIAGEN’s licensed software Ingenuity® Pathway Analysis (IPA®, QIAGEN Redwood City,CA, USA, www.qiagen.com/ingenuity) with the default options for pathway analysis.

#### Database search

We used the Ensembl genome browser (https://www.ensembl.org/index.html, release 90, August 2017), National Center for Biotechnology Information (NCBI), the Rat Genome Database (RGD; http://rgd.mcw.edu), and UniProt (http://www.uniprot.org/) for the analysis of gene functions and associated medical conditions (phenotypes). Among the phenotypes, we focused primarily on the following keywords: a) sleep, b) syndrome, and post-hoc we also added c) visual/retinal abnormalities.

#### Gene expression analysis

The correlation analyses of M-values with corresponding gene expression were performed in SPSS (IBM Corp. Released 2016. IBM SPSS Statistics for Windows, Version 24.0. Armonk, NY: IBM Corp.), using Pearson correlation.

#### Sampling time

The correlation analyses of M-values with sample time were conducted in SPSS, using Pearson correlation.

#### Study approval

Sample collection and study design for DILGOM and Airline were performed according to the principles of the Declaration of Helsinki and were approved by Coordinating Ethics Committee of the Helsinki and Uusimaa Hospital District. All participants provided written informed consent.

## Supplementary information

Supplementary Information

## Data Availability

The ethical approval limits the individual-level data availability from Airline and DILGOM cohorts, and prohibits the authors from making the data set publicly available. Data are available from the corresponding author (Tiina Paunio) upon ethical approval from the Coordinating Ethics Committee of the Helsinki and Uusimaa Hospital District.
